# Role of ERK/MAPK in endothelin receptor signaling in human aortic smooth muscle cells

**DOI:** 10.1186/1471-2121-10-52

**Published:** 2009-07-03

**Authors:** Qing-wen Chen, Lars Edvinsson, Cang-Bao Xu

**Affiliations:** 1 Division of Experimental Vascular Research, Institute of Clinical Science in Lund, Lund University, Lund, Sweden; 2 Department of Clinical and Experimental Research, Glostrup Hospital, Copenhagen University, Copenhagen, Denmark

## Abstract

**Background:**

Endothelin-1 (ET-1) is a potent vasoactive peptide, which induces vasoconstriction and proliferation in vascular smooth muscle cells (VSMCs) through activation of endothelin type A (ET_A_) and type B (ET_B_) receptors. The extracellular signal-regulated kinase 1 and 2 (ERK1/2) mitogen-activated protein kinases (MAPK) are involved in ET-1-induced VSMC contraction and proliferation. This study was designed to investigate the ET_A _and ET_B _receptor intracellular signaling in human VSMCs and used phosphorylation (activation) of ERK1/2 as a functional signal molecule for endothelin receptor activity.

**Results:**

Subconfluent human VSMCs were stimulated by ET-1 at different concentrations (1 nM-1 μM). The activation of ERK1/2 was examined by immunofluorescence, Western blot and phosphoELISA using specific antibody against phosphorylated ERK1/2 protein. ET-1 induced a concentration- and time- dependent activation of ERK1/2 with a maximal effect at 10 min. It declined to baseline level at 30 min. The ET-1-induced activation of ERK1/2 was completely abolished by MEK1/2 inhibitors U0126 and SL327, and partially inhibited by the MEK1 inhibitor PD98059. A dual endothelin receptor antagonist bosentan or the ET_A _antagonist BQ123 blocked the ET-1 effect, while the ET_B _antagonist BQ788 had no significant effect. However, a selective ET_B _receptor agonist, Sarafotoxin 6c (S6c) caused a time-dependent ERK1/2 activation with a maximal effect by less than 20% of the ET-1-induced activation of ERK1/2. Increase in bosentan concentration up to 10 μM further inhibited ET-1-induced activation of ERK1/2 and had a stronger inhibitory effect than BQ123 or the combined use of BQ123 and BQ788. To further explore ET-1 intracellular signaling, PKC inhibitors (staurosporin and GF109203X), PKC-delta inhibitor (rottlerin), PKA inhibitor (H-89), and phosphatidylinositol 3-kinase (PI3K) inhibitor (wortmannin) were applied. The inhibitors showed significant inhibitory effects on ET-1-induced activation of ERK1/2. However, blockage of L-type Ca^2+ ^channels or calcium/calmodulin-dependent protein kinase II, chelating extracellular Ca^2+ ^or emptying internal Ca^2+ ^stores, did not affect ET-1-induced activation of ERK1/2.

**Conclusion:**

The ET_A _receptors predominate in the ET-1-induced activation of ERK1/2 in human VSMCs, which associates with increments in intracellular PKC, PKA and PI3K activities, but not Ca^2+ ^signalling.

## Background

In the human cardiovascular system, endothelin-1 (ET-1) is the most important isoform, which induces long-lasting vasoconstriction and stimulates proliferation of vascular smooth muscle cells (VSMCs) [1]. ET-1 acts on two G-protein coupled receptors: endothelin type A (ET_A_) and endothelin type B (ET_B_), and plays an important role in hypertension, vascular remodelling, cardiac hypertrophy and coronary artery disease [2]. The ET_A _receptors locate on VSMCs and mediate vasoconstriction, while the ET_B _receptors primarily locate in vascular endothelial cells and mediate transient vasodilation *in vivo *[3]. However, a subpopulation of contractile ET_B _receptors exist in the VSMCs and mediate vasoconstriction [3,4]. The ET_A _receptor activates G proteins of Gq/11 and G12/13, which results in the contractile and proliferation effects in VSMCs through activation of diverse signaling molecules such as phospholipase C (PLC), intracellular Ca^2+^, protein kinase C (PKC), and extracellular signal-regulated kinase 1 and 2 (ERK1/2). Whereas, the ET_B _receptor stimulates the Gi and the Gq/11 families in VSMCs and endothelial cells [1,2,5,6]. ET-1 is non-selective agonist for both ET_A _and ET_B _receptors, which may result in receptor signal cross-talk in vascular physiology and pathology. However, there is limited knowledge about this.

ERK1/2, also termed p44/42 MAPK (mitogen-activated protein kinase), is one of the members of MAPK superfamily, which includes a family of serine/threonine kinase associated with VSMCs contraction, proliferation, migration, differentiation, adhesion, collagen deposition and survival [7]. Activation of either the ET_A _or the ET_B _receptor results in phosphorylation of ERK1/2, which is an important regulator for cellular proliferation, migration, differentiation and vascular smooth muscle constriction [8-12]. A MAPK kinase (MEK) is required for the ERK1/2 phosphorylation of both threonine and tyrosine residues [13]. In the activated form, ERK1/2 transmits extracellular stimuli by phosphorylating a variety of substrates including transcription factors and kinases. There is a paucity of knowledge on intracellular signal mechanisms that ET-1 leads to activation of ERK1/2 in human VSMCs. Non-receptor tyrosine kinase c-Src-independent small G protein Ras-Raf-dependent mechanisms have been reported to mediate ET-1-induced ERK1/2 phosphorylation in cultured mouse VSMCs [14]. Intracellular Ca^2+ ^signals are required for MAPK/ERK1/2 activation induced by angiotensin II in VSMCs [15-17]. However, ET-1-induced vasoconstriction is not affected by calcium channel blockers [18]. Thus, Ca^2+^-independent contraction is suggested to be associated with PKC, phosphoinositide 3-kinase (PI3K), Rho kinase and MAPK [10,11,19]. The present study was designed, by using a series of specific pharmacological inhibitors, to explore the intracellular signal mechanisms that ET-1 leads to activation of ERK1/2 in human VSMCs with special focus on the receptor signalling. We have demonstrated that ET_A _receptors predominate over ET_B _receptors in mediating ET-1-induced activation of ERK1/2 in human VSMCs. This activation is associated with PKC, PKA and PI3K activities, but not intracellular Ca^2+ ^signalling.

## Results

### Time course and concentration-dependent activation of ERK1/2 induced by ET-1

**Figure 1:**
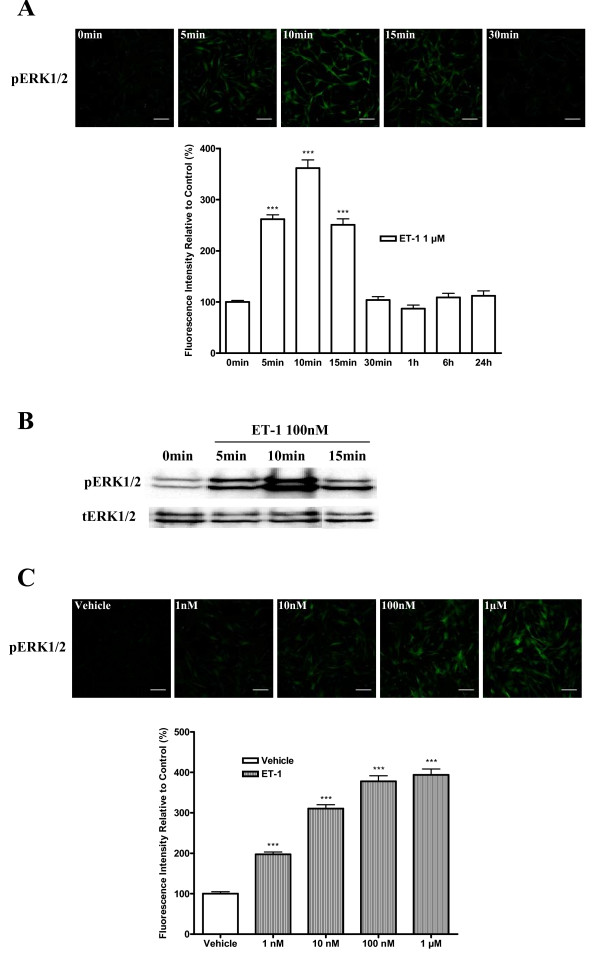
**Time course and concentration-effects of ET-1 on activation of ERK1/2**. Cultured HASMCs were starved for 24 h in serum-free medium and then stimulated with ET-1. A, bar graph shows time-dependent activation of ERK1/2 by ET-1 at 1 μM. Phosphorylated ERK1/2 was determined by immunofluorescence with an anti-phospho-ERK1/2 antibody. B, representative autoradiograph of western blot showing the level of phosphorylated and total ERK1/2 from the samples treated with ET-1 at 0 min, 5 min,10 min and 15 min. C, Bar graph shows concentration-dependent activation of ERK1/2 by ET-1 from 1 nM to 1 μM for 10 min. Phosphorylated ERK1/2 was determined by immunofluorescence with an anti-phospho-ERK1/2 antibody. The upper panels of A and C indicate representative images of immunofluorescence showing the phosphorylated ERK1/2 from the samples treated with ET-1 at different time points and various concentrations, respectively. The scale bar in each image represents 20 μm. Data represent mean ± S.E.M. *** p < 0.001 compared with the vehicle value. p = phosphorylated; t = total.

ET-1-induced activation of ERK1/2 was examined in human aortic smooth muscle cells (HASMCs) at different time points and ET-1 concentrations. There was a 2.6 fold (p < 0.001) increase of phosphorylated ERK1/2 in cells exposed to 1 μM of ET-1 for 5 min; the enhancement reached a peak (3.6 fold, p < 0.001) at 10 min after exposure to ET-1 (Figure 1A). Thereafter, the activities of ERK1/2 induced by ET-1 rapidly declined, and returned to baseline control value at 30 min after stimulation. As verified by western blot (Figure 1B), there was an increase in pERK1/2 after ET-1 treatment. The concentration effects of ET-1 on ERK1/2 activation were investigated at 10 min. It showed that ET-1 induced activation of ERK1/2 in a concentration-dependent manner from 1 nM to 1 μM (Figure 1C).

### Roles of endothelin receptors in mediating ET-1-induced activation of ERK1/2

**Figure 2:**
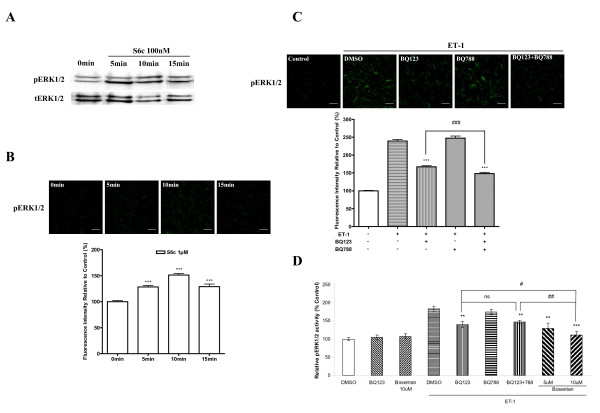
**Roles of endothelin receptor subtypes in mediating ET-1-induced activation of ERK1/2 in HASMCs**. Serum-starved cells were stimulated with S6c for 5, 10 or 15 min or ET-1 for 10 min. 5 μM of BQ123, 5 μM of BQ788, 5 μM or 10 μM of bosentan were given for 30 min before addition of ET-1. A, representative autoradiograph of western blot showing the phosphorylated ERK1/2 and total ERK1/2 from samples treated with 100 nM of S6c at different time points. B, bar graph shows time-dependent activation of ERK1/2 by 1 μM of S6c. Phosphorylated ERK1/2 was determined by immunofluorescence with an anti-phospho-ERK1/2 antibody. C, bar graph shows inhibitory effects of ET receptor inhibitors on phosphorylated ERK1/2 induced by 10 nM of ET-1. Phosphorylated ERK1/2 was determined by immunofluorescence with an anti-phospho-ERK1/2 antibody. D, inhibitory effects of ET receptor inhibitors on phosphorylated ERK1/2 activity induced by 10 nM of ET-1. Phosphorylated ERK1/2 activity was determined by phosphoELISA assay as described in Methods. The upper panels of B and C indicate representative images of immunofluorescence showing the phosphorylated ERK1/2 from samples treated with S6c at different time points and treated with ET receptor inhibitors prior to addition of ET-1, respectively. The scale bar in each image represents 20 μm. Data represent mean ± S.E.M. *** p < 0.001 compared with the vehicle value (B). ** p < 0.01, *** p < 0.001 compared with the ET-1-stimulated states after DMSO treatment (C,D). # p < 0.05, ## p < 0.01, ### p < 0.001; p = phosphorylated; t = total, ns = non-significant.

The roles of ET_A _and ET_B _receptors in mediating ET-1-induced activation of ERK1/2 were studied by using bosentan (a dual endothelin receptor antagonist), BQ123 (a selective peptide antagonist for the ET_A _receptor), and BQ788 (a selective peptide antagonist for the ET_B _receptor). To clarify if the ET_B _receptors in HASMCs were involved in ET-1- induced activation of ERK1/2, sarafotoxin 6c (S6c), a selective ET_B _receptor agonist was employed and the phosphorylation of ERK1/2 was examined by immunofluorescence and western blot (Figure 2B and 2A). In figure 2B, there was a slight elevation of phosphorylated ERK1/2 (1.3 fold, p < 0.001) as observed at 5 min after exposure to 1 μM of S6c. This peaked at 10 min (1.5 fold, p < 0.001), and quickly declined at 15 min (1.3 fold, p < 0.001). This slight transient increase of phosphorylated ERK1/2 was also produced by 100 nM of S6c and verified by western blot for pERK1/2 (Figure 2A). BQ123 and bosentan significantly inhibited the increase in pERK1/2 activities, while the ET_B _receptor antagonist BQ788 had no significant effect (Figure 2C and 2D). The increase in phosphorylated ERK1/2 was significantly inhibited by 5 μM of BQ123 (by 51.8%, Figure 2C), which is consistent with the results of phosphoELISA assay (by 51.9%, Figure 2D) and western blot (by 56.2%) [see Additional file 1]. ET-1-induced ERK1/2 activation was also significantly inhibited by combination of BQ123 and BQ788 by 65.4% (Figure 2C in immunocytochemistry), by 43.6% (Figure 2D in phophoELISA assay) and by 62.1% [see Additional file 1 in western blot]. Compared to BQ123, a further inhibitory effect was seen in combination of BQ123 and BQ788 (p < 0.001, Figure 2C). Bosentan at 5 μM and 10 μM significantly inhibited ET-1- induced activation of ERK1/2 by 65.1% and 87.1%, respectively (Figure 2D). At 10 μM bosentan had a stronger inhibitory effect on ET-1-induced activation of ERK1/2 than either BQ123 or combination of BQ123 and BQ788 (p < 0.05, or p < 0.01, Figure 2D). This indicated that ET_B _receptor antagonist BQ788 had no significant inhibitory effect on ET-1-induced activation of ERK1/2 in the absence of ET_A _receptor antagonist BQ123, while bosentan, a dual ET receptor agonist or combined use of BQ123 and BQ788, further decreased ET-1-induced activation of ERK1/2.

### Role of the MEK on ET-1-induced activation of ERK1/2

**Figure 3:**
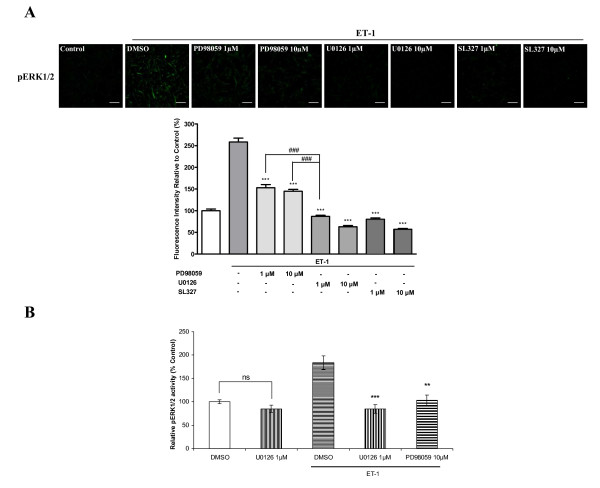
**Effects of MEK inhibitors on ET-1-induced activation of ERK1/2 in HASMCs**. Serum-starved cells were treated with U0126, PD98059 or SL327 for 30 min prior to addition of ET-1. A, bar graph shows inhibitory effects of MEK inhibitors on phosphorylated ERK1/2 induced by 10 nM of ET-1. Phosphorylated ERK1/2 was determined by immunofluorescence with an anti-phospho-ERK1/2 antibody. B, inhibitory effects of MEK inhibitors on phosphorylated ERK1/2 activity induced by 10 nM of ET-1. Phosphorylated ERK1/2 activity was determined by phosphoELISA assay as described in Methods. The upper panel of A indicates representative images of immunofluorescence showing the phosphorylated ERK1/2 from the samples treated with MEK inhibitors prior to addition of ET-1. The scale bar in each image represents 20 μm. Data represent mean ± S.E.M. ** p < 0.01, *** p < 0.001 compared with the ET-1-stimulated states after DMSO treatment. ### p < 0.001; p = phosphorylation, ns = non-significant.

Three different MEK/ERK kinase inhibitors were used to study ET-1-induced activation of ERK1/2 in HASMCs. As shown in Figure 3A and 3B, U0126, a potent MEK1/2 inhibitor, at the concentration 1 and 10 μM completely inhibited ET-1-induced phosphorylation of ERK1/2 from 258% to 87% and 63%, respectively. SL327, another selective inhibitor of MEK1 and MEK2 had similar degree of inhibitory effects (Figure 3A). PD98059, a selective inhibitor of MEK1, only partially inhibited ET-1-induced phosphorylation of ERK1/2 from 258% to 153% at 1 μM, and to 145% at 10 μM, respectively (Figure 3A). This suggests that both MEK1 and MEK2 are required for ET-1 to activate ERK1/2 in HASMCs. This is further supported by phosphoELISA assay (Figure 3B) and western blot [see Additional file 1]. Compared to PD98059, U0126 at 1 μM had a significant stronger inhibitory effect (p < 0.001, Figure 3A). To clarify whether U0126 also inhibits phosphorylation of ERK1/2 in untreated control cells, the phosphoELISA assay was used. It showed that in untreated control HASMCs, U0126 at 1 μM did not significantly modify ERK1/2 activity (Figure 3B). In ET-1-treated HASMCs, U0126 significantly decreased the phosphorylated ERK1/2 level at the same concentration (Figure 3A and 3B).

### Roles of PKC/PKA and small G proteins on ET-1-induced activation of ERK1/2

**Figure 4:**
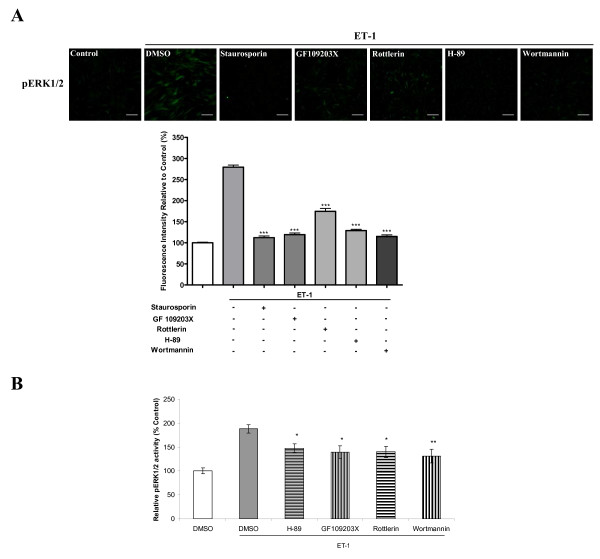
**Effects of PKC, PKA and PI3 kinase inhibitors on ET-1-induced activation of ERK1/2 in HASMCs**. Serum-starved cells were treated with 500 nM of staurosporin, 10 μM of GF109203X, 5 μM of rottlerin, 10 μM of H-89, or 2 μM of wortmannin for 30 min prior to addition of ET-1. A, bar graph shows inhibitory effects of inhibitors on phosphorylated ERK1/2 induced by 10 nM of ET-1. Phosphorylated ERK1/2 was determined by immunofluorescence with an anti-phospho-ERK1/2 antibody. B, inhibitory effects of inhibitors on phosphorylated ERK1/2 activity induced by 10 nM of ET-1. Phosphorylated ERK1/2 activity was determined by phosphoELISA assay as described in Methods. The upper panel of A indicates representative images of immunofluorescence showing the phosphorylated ERK1/2 from samples treated with inhibitors prior to addition of ET-1. The scale bar in each image represents 20 μm. Data represent mean ± S.E.M. * p < 0.05, **p < 0.01, *** p < 0.001 compared with the ET-1-stimulated states after DMSO treatment. p = phosphorylation.

To further determine the upstream signaling involved in the MEK/ERK pathway, we used pharmacological inhibitors and examined the effects of PKC inhibitors (staurosporin and GF109203X), PKC-delta inhibitor (Rottlerin), PKA specific inhibitor (H-89), and PI3K inhibitor (wortmannin) on ET-1-induced pERK1/2 activities (Figure 4). The activation of ERK1/2 was significantly inhibited by 500 nM of staurosporin (93.2%), 10 μM of GF 109203X (89.1%), 5 μM of Rottlerin (58.4%), 10 μM of H-89 (83.8%), and 2 μM of Wortmannin (91.6%), respectively (Figure 4A). Similar, results were obtained in the phosphoELISA assay (Figure 4B).

### Role of extracellular Ca^2+ ^influx or intracellular Ca^2+ ^release in mediating ET-1-induced activation of ERK1/2 in HASMCs

**Figure 5:**
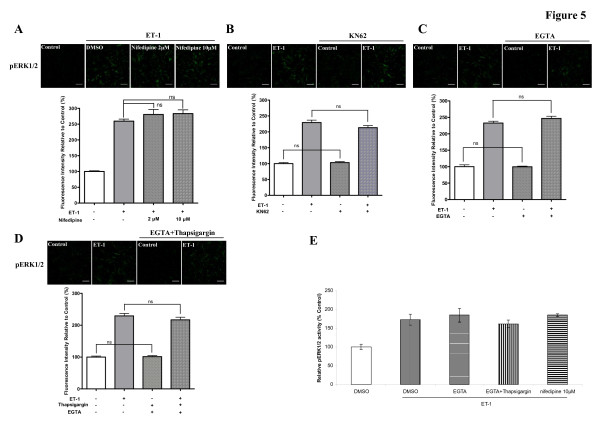
**Role of intracellular Ca^2+ ^in mediating ET-1-induced activation of ERK1/2 in HASMCs**. Serum-starved cells were treated with 10 nM of ET-1 for 10 min after different treatments. A, L-type Ca^2+ ^channel inhibitor nifedipine was treated for 30 min before addition of ET-1, bar graph shows effects of nifedipine at 2 μM and 10 μM on phosphorylated ERK1/2 induced by ET-1. Phosphorylated ERK1/2 was determined by immunofluorescence with an anti-phospho-ERK1/2 antibody. B, 10 μM of KN-62 was given for 30 min before addition of ET-1, bar graph shows effect of KN-62 on phosphorylated ERK1/2 induced by ET-1. C, 5 mM of the Ca^2+ ^chelator EGTA was administered 15 min before addition of ET-1, bar graph shows effect of EGTA on phosphorylated ERK1/2 induced by ET-1. D, the cells were treated with 1 μM of thapsigargin with 5 mM of EGTA for 15 min before addition of ET-1, bar graph shows effect of thapsigargin on phosphorylated ERK1/2 induced by ET-1 in the presence of EGTA. E, the treatment of cells with 10 μM of nifedipine, 5 mM of EGTA, or 1 μM of thapsigargin with 5 mM of EGTA before addition of ET-1, bar graph shows effects of the different treatments on phosphorylated ERK1/2 activity induced by ET-1 as determined by the phosphoELISA assay. The upper panels of A, B, C and D indicate representative images of immunofluorescence illustrating the phosphorylated ERK1/2 from samples given the different treatments prior to addition of ET-1. The scale bar in each image represents 20 μm. Data represent mean ± S.E.M. ns = non-significant. p = phosphorylation.

Ca^2+^, a second messenger, has a central role in activation of various key cellular responses, including muscle contraction, cell proliferation, migration and adhesion [20]. To evaluate the role of intracellular Ca^2+^signaling in mediating ET-1-induced activation of ERK1/2, nifedipine was used to block external Ca^2+ ^influx through L-type Ca^2+ ^channels, 5 mM of EGTA was employed to chelate extracellular Ca^2+^, and 1 μM of thapsigargin was used to cause intracellular Ca^2+ ^stores to become depleted. KN-62, a calcium-calmodulin dependent protein kinase II (CAMKII) inhibitor was also examined (Figure 5). The activation of ERK1/2 was not affected by L-type Ca^2+ ^channel blocker (Figure 5A), chelating extracellular Ca^2+ ^(Figure 5C), abolishing intracellular Ca^2+ ^release (Figure 5D), or inhibition of CAMKII (Figure 5B). Replacing the medium with calcium-free PBS [see Additional file 2] did not inhibit ET-1-induced activation of ERK1/2. These indicated that extracellular Ca^2+ ^influx and Ca^2+ ^released from internal stores were not necessarily required for the ET-1-induced phosphorylation of ERK1/2 in HASMCs. This is further supported by the results from phosphoELISA assay (Figure 5E). To identify whether extracellular Ca^2+ ^was chelated or Ca^2+ ^influx was decreased in our experiments, we used 1 μM of thapsigargin to induce extracellular Ca^2+ ^influx through store-operated Ca^2+ ^channels (SOCC) [21]. We found that thapsigargin resulted in an activation of ERK1/2 in HASMCs as reported in RBL-1 cells [21]. The activation of ERK1/2 was abolished by 5 mM of EGTA [see Additional file 3]. This suggests that 5 mM of EGTA can effectively chelate extracellular Ca^2+^and decrease Ca^2+ ^influx in our experiments.

## Discussion

The present study has revealed that ET-1 acts primarily via the ET_A _receptors to induce phosphorylation of ERK1/2 in HASMCs. The ET-1-induced response requires intracellular signal molecule PKC, PKA and PI3K activities, while it is independent of intracellular calcium signaling.

### ET-1-induced activation of ERK1/2 in HASMCs

ERK1/2 are important regulators of cell proliferation and migration in VSMCs [8,9]. These basic cellular functions are important for the formation of the neointima in pathologic states such as atherosclerosis. Many stimuli such as mechanical stretch, growth factors, cytokines and activation of G protein-coupled receptors, can result in phosphorylation of ERK1/2 and its signal pathways. Recent studies have demonstrated that ERK1/2 MAPK pathways regulate Ca^2+^-dependent and Ca^2+^-independent contraction of VSMCs [10-12,19]. Intracellular ERK1/2 MAPK signal mechanisms play important roles in vascular pathology and in the development of cardiovascular disease [22-24]. ET-1 not only remains the most potent and long-lasting vasoconstrictor of human vessels, it also induces proliferation of vascular smooth muscle cells through activation of ERK1/2 [25] in pulmonary hypertension, atherosclerosis, heart failure and restenosis [2,26]. In human arterial smooth muscle cells, ET-1-induced activation of ERK1/2 is much weaker in aortic artery than in coronary artery [27]. This implies that small arteries are more sensitive than large arteries. Unlike angiotensin II, which shows a rapid and transient increase in activities of ERK1/2 [14], ET-1 induced a long-lasting phosphorylation of ERK1/2 with a peaked at 10 min and declined to baseline after 30 min in present study. The activation of ERK1/2 by ET-1 might contribute to VSMC proliferation in formation of new intima and thus it may contribute to serve as an early "switch-on" mechanism for cardiovascular disease development [28].

### Roles of ET receptors in activation of ERK1/2 in HASMCs

The physiological and pathological effects of ET-1 are mediated through two G protein-coupled receptors, ET_A _and ET_B_. In human vasculature, ET_A _receptors predominate on the smooth muscle cells and mediate constriction, whereas ET_B _receptors are expressed less than 15% on these cells [29,30]. *In-vivo *studies suggest that both subtypes of endothelin receptors can mediate vasoconstriction in human resistance and capacitance vessels [31]. In the present study, we found that ET_A _predominately mediated ET-1-induced activation of ERK1/2. Although some activation of ERK1/2 was obtained with the ET_B_-selective agonist, S6c, the maximum response produced to S6c was transient and less than 20% of the ET-1 effect. In addition, BQ123, a selective antagonist of the ET_A _receptor [32], but not ET_B _receptor antagonist BQ788, significantly inhibited the activation of ERK1/2 induced by ET-1, suggesting that ET-1-induced activation of ERK1/2 is predominately mediated by ET_A_receptors. Compared to BQ123, a further inhibition of ET-1-induced activation of ERK1/2 was obtained in combination of BQ123 and BQ788. Bosentan, a dual ET_A _and ET_B _receptor antagonist had a significant stronger inhibitory effect on ET-1-induced activation of ERK1/2 than either BQ123 or the combination of BQ123 and BQ788. These results suggest that ET receptor dimerization [33] might also occur in human VSMCs in the presence of ET-1 as a bivalent ligand connecting two receptors [34-36] and that the receptor cross-talk is involved in the ET-1 effect. However, this requires more studies to verify.

### Upstream intracellular signal molecules involved in ET-1-induced activation of ERK1/2

ERK1/2 activation requires a sequential activation of Ras, Raf and MEK signal cascades [14,37]. MEK inhibitors (U0126, PD98059 and SL327) were used to investigate the role of upstream MEK in ET-1-induced activation of ERK1/2. U0126, a highly selective inhibitor of MEK1/2 had the same potency as SL327 (another selective inhibitor of MEK1/2), and completely inhibited ET-1-induced activation of ERK1/2, whereas, PD98059, a selective MEK1 inhibitor, only partially inhibited ET-1-induced activation of ERK1/2. PKC, a family of serine/threonine kinases, may be involved in the intracellular signal transduction of MEK/ERK1/2 induced by ET-1. PKA is an important second messenger. Cyclic AMP-independent activation of PKA by ET-1 has been observed in rat aortic smooth muscle cells [38]. On the other hand, G-protein-coupled receptor signaling can be mediated through various small G proteins. The Ras/Raf pathway is found to be a proximal regulator of MEK [14,39]. PI3K, another downstream effector of Ras [40], has been linked to a diverse group of cellular functions, including cell growth, proliferation, differentiation, motility, survival [41]. By using selective inhibitors, the present study revealed that PKC, PKA and PI3K were involved in activation of ERK1/2 induced by ET-1 in HASMCs, which may provide targets for drug discovery [42].

### Intracellular Ca^2+ ^signaling was not required for ET-1-induced activation of ERK1/2

ET-1 stimulates phospholipase C-dependent hydrolysis of PIP_2 _(phosphatidylinositol 4,5-bisphosphate) through G-protein coupled receptors, leading to the generation of inositol 1,4,5-trisphosphate (IP3) and diacylglycerol (DAG), which are involved in intracellular Ca^2+ ^mobilization and PKC activation [5]. Recently, growing evidence has shown that Ca^2+ ^signaling is critical for activation of ERK1/2 induced by angiotensin II in VSMCs [15-17]. However, the role of intracellular Ca^2+ ^signaling in ET-1-induced activation of ERK1/2 in human VSMCs remains unclear. It has been reported that the activation of L-type Ca^2+ ^channels contributes to ET-1-induced sustained phase of the Ca^2+ ^response and the ability to generate force [43]. Unlike angiotensin II, the present study revealed that extracellular Ca^2+ ^influx through L-type Ca^2+ ^channels did not participate in ET-1-induced activation of ERK1/2 in human VSMCs. To further investigate the involvement of intracellular Ca^2+ ^through other Ca^2+ ^channels, which are suggested to be involved in ET-1-mediated contractions of VSMC [43] and mitogenesis [44], 5 mM of EGTA was used. Extracellular Ca^2+ ^chelation by EGTA did not affect activation of ERK1/2 induced by ET-1. ET-1-induced Ca^2+ ^release from intracellular stores is triggered by the binding of IP3 to receptors on the sarcoplasmic reticulum (SR). Depletion of intracellular Ca^2+ ^stores can lead to a local Ca^2+ ^flux through store-operated Ca^2+ ^channels (SOCC), which has been reported to initiate the activation of ERK1/2 in RBL-1 cells [21]. Therefore, in our studies, thapsigargin, an inhibitor to the SR Ca^2+^-ATPase pump, which results in Ca^2+ ^release and depletion from internal stores, was applied together with 5 mM of EGTA. The results showed that ERK1/2 activation by ET-1 did not require the participation of intracellular Ca^2+ ^release. Studies have indicated that the CAMKII pathway mediates G-protein coupled receptor ligand-depedent activation of ERK1/2 in cultured VSM cells [36,45,46]. However, we observed that CAMKII pathway was probably not involved in the ET-1- induced activation of ERK1/2 in human VSMCs as based on KN-62 inhibition experiment. Using receptor-operated Ca^2+ ^channel blockers LOE 908 and SK&F 96365, and L-type Ca^2+ ^channels blocker nifedipine, Kawanabe et al noted that ET-1-induced ERK1/2 activiation involved a Ca^2+ ^influx-dependent cascade through Ca^2+^permeable nonselective cation channels (NSCCs) and SOCC, and a Ca^2+^influx-independent cascade in rabbit carotid artery VSMCs [47]. The studies showed that maximal effective concentration of nifedipine has only 10% of the inhibition on ET-1-induced increases in ERK1/2 activity. However, we did not find significant changes of phosphorylated ERK1/2 induced by ET-1 after treatment with nifedipine or chelation of extracellular Ca^2+^.

## Conclusion

In conclusion, we have demontrated that ET-1-induced activation of ERK1/2 in human VSMCs is predominantly mediated by ET_A _receptors through upstream signal molecule PKC, PKA and PI3K, while it is independent of CAMKII and intracellular Ca^2+ ^signaling. The endothelin system plays key roles in hypertension, stoke and myocardial infarction. Understanding the intracellular signaling mechanisms of endothelin receptors may provide new strategies for developing new drugs for cardiovascular diseases.

## Methods

### Reagents and antibodies

ET-1 and S6c, a selective ET_B _receptor agonist [48], were used at different concentration to stimulate phosphorylation (activation) of ERK1/2 in human VSMCs. To detect the intracellular signal pathways involved in activation of ERK1/2, a set of inhibitors were administered prior to addition of stimulators. Bosentan, a dual endothelin receptor antagonist was purchased from SynFine Research (Ontario, Canada). ET_A _antagonist BQ123 and ET_B _antagonist BQ788 [4,48] were employed to examine the mediation of endothelin receptors in activation of ERK1/2. PD98059, a MEK1 inhibitor, and U0126, SL327, selective inhibitors of both MEK1 and MEK2, were used as ERK inhibitors. Staurosporin and GF109203X, PKC inhibitors; Rottlerin, a PKC-delta inhibitor; H-89, a PKA inhibitor; Wortmannin, a specific inhibitor of PI3K, were used as protein kinase inhibitors or phosphoinositide 3-kinase inhibitor. Nifedipine, a L-type Ca^2+ ^channels inhibitor; EGTA (ethylene glycol tetraacetic acid), a Ca^2+ ^chelator; thapsigargin, a sarco-endoplasmic reticulum Ca^2+^-ATPase pump inhibitor; KN-62, a CAMKII inhibitor, were applied to determine the involvement of Ca^2+ ^signaling and CAMKII in activation of ERK1/2. The concentration of inhibitors was determined by recommendation from product data sheet and literatures. All drugs were purchased from Sigma-Aldrich Co. (St. Louis, MO, USA). ET-1 and S6c were dissolved in sterile water with 0.1% BSA; the other reagents were dissolved in DMSO as a stock solution and diluted in cell culture medium before use.

A monoclonal antibody for phospho-ERK1/2 (phospho T183 + Y185) and a polyclonal antibody for total-ERK1/2 were obtained from Abcam plc. (Cambridge, UK). Polyclonal β-actin was purchased from Cell Signaling Technology, Inc. (Boston, MA, USA).

### Cell Culture and Experimental Protocol

HASMCs at the end of the tertiary culture stage were obtained as a commercially available product from Cascade Biologics Inc. (Portland, OR, USA). Cells were plated in 75 cm2 tissue culture flasks at a density of 2.5 × 103 viable cells/cm2 in Medium 231 supplemented with 5% smooth muscle growth supplement (SMGS). Medium 231 and SMGS were purchased from Cascade Biologics Inc. The cells were incubated in a 5% CO2 incubator at 37°C and the medium was replaced every other day until the culture was approximately 80–90% confluent. Then the cells were removed from the flasks with accutaseTM Enzyme Cell Detachment Medium (eBioscience, Inc. San Diego, CA, USA) and seeded onto 100-mm tissue culture dish (Greiner Bio-One GmbH, Frickenhausen, Germany).

All experiments were performed with the cells of passages 6 to 9. HASMCs were allowed to grow to 70%–80% confluence within 2 to 3 days, and maintained in medium 231 with 0.05% SMGS for 24 h, then we added vehicle or ET-1, S6c at different concentration from 1 nM to 1 uM, or with a time course at 5 min, 10 min, 15 min, 30 min, 1 h, 6 h and 24 h. Inhibitors or DMSO were treated for 30 min prior to addition of ET-1.

### Immunofluorescence Analysis to Detect phosphorylated ERK1/2

HASMCs were seeded at a density of 5 × 10^3^/well in 4 well NUNC Lab-Tek II Chamber Slides for 3 days and were starved in medium 231 with 0.05% SMGS for 24 h. The cells were stimulated with ET-1 or S6c at above indicated time points after treatment with vehicle or inhibitors for 30 minutes, and then washed, fixed in 4% paraformaldehyde, permeabilized in PBS containing 4% Triton X-100. The monoclonal primary antibody against phospho-ERK1/2 (phospho T183 + Y185) was added to the cells at 1: 1000 dilution and incubated at room temperature for 1 h or overnight at 4°C, followed by adding fluorescein isothiocynate (FITC)-conjugated goat anti-mouse secondary antibody at 1:5000 dilution in dark according to the recommendation of the manufacturer. In the control experiments, either the primary antibody or the secondary antibody was omitted. After washing with PBS, ProLong Gold antifade mounting reagent (Invitrogen Corporation, Carlsbad, CA, USA) was added and the cells were sealed with cover slip on the slide. The immunofluorescence stained cells were observed under a laser scanning confocal microscope (Nikon, C1plus, Nikon Instruments Inc., NY, USA) and analysed by ImageJ software . The fluorescence intensity of cells was measured at 4 preset areas of per sample and at least three independent experiments were performed. The fluorescence intensity of each treated group was determined as the percent increase over control, with the control normalized to 100%. There was no change of fluorescence intensity after cells were treated with inhibitors compared with vehicle treatment [see Additional file 4].

### Western Blot Analysis

About 70%–80% confluent HASMCs in 100-mm tissue culture dishes were made quiescent by placing them in medium 231 supplemented with 0.05% SMGS for 24 h and harvested in cell extract denaturing buffer (BioSource, USA) with addition of a phosphatase inhibitor cocktail and protease inhibitor cocktail (Sigma, USA) after treatment. Incubating cells at 4°C for 30 min, whole cell lysates were sonicated for 2 min on ice, centrifuged at 15,000 × g at 4°C for 30 min, and the supernatants were collected as protein samples. The protein concentrations were determined using the protein assay reagents (Bio-Rad, Hercules, CA, USA) and stored at -80°C until immunoblotting assay. The protein homogenates were diluted 1:1(v/v) with 2 × SDS sample buffer (Bio-Rad, USA). 25–50 ug of total proteins were boiled for 10 min in SDS sample buffer and separated by 4–15% SDS Ready Gel Precast Gels (Bio-Rad, USA) for 120 min at 100 v, and transferred electrophoretically to nitrocellulose membranes (Bio-Rad, USA) at 100 v for 60 min. The membrane was then blocked for 1 h at room temperature with phosphate buffered saline (PBS) containing 0.1% Tween-20 (Sigma, USA) and 5% non-fat dried milk, and incubated with primary antibodies diluted 1:1000 overnight at 4°C, followed by incubation with ECL anti-mouse or anti-rabbit IgG, horseradish peroxidase-conjugated secondary antibodies (Amersham Biosciences, Piscataway, NJ, USA) diluted 1:10000 for 1 h at room temperature. The probed proteins were developed by LumiSensor Chemiluminescent HRP Substrate ECL Western Blot Detection Reagent (GenScript Corp., Piscataway, NJ, USA). To detect multiple signals using a single membrane, the membrane was incubated for 5–15 min at room temperature with restore plus western blot stripping buffer (Pierce Biotechnology, Inc., Rockford, IL. USA). The membranes were visualized using a Fujifilm LAS-1000 Luminiscent Image Analyzer (Stamford, CT, USA), and then quantification of band intensity was analyzed with Image Gauge Ver. 4.0 (Fuji Photo Film Co., LTD., Japan). Three independent experiments were performed in duplicate.

### Cell-based PhosphoELISA Analysis

HASMCs were seeded at a density of 3 × 10^3^/well in 96-well plate for 3 days and starved in medium 231 with 0.05% SMGS for 24 h. The cells were treated with vehicle or different inhibitors for 30 min prior to the addition of ET-1. After 10 min of ET-1 stimulation, the cells were fixed and stored at 4°C until the performance of experiments. Phosphorylated ERK1/2 was measured using a cell-based ELISA Assay Kit (SABiosciences Corporation, MD, USA) following the manufacturer's instructions. Phosphorylated ERK1/2 activity was presented as a relative extent to the level of total ERK1/2. Independent experiments were done in duplicate or triplicate and were repeated at least three times.

### Statistical Analysis

Comparison between two groups was performed using two-tailed unpaired Student's t-test with Welch's correction. For more than two groups one-way ANOVA followed by Dunnett's post test was used. A p-value, less than 0.05 was considered to be significant. Results were presented as mean ± SEM. At least 3 different samples or independent experiments were analyzed in each group.

## Authors' contributions

QC carried out the main part of the experiments, participated in the design, statistical analysis, drafting and writing of the manuscript. LE participated in the writing of the manuscript. CX conceived the study and the design, coordinated the work and the writing of the manuscript. All authors have read and approved the final manuscript.

## Supplementary Material

Additional file 1 **Inhibitory effects of BQ123 and U0126 on pERK1/2 activity in HASMCs**. The data provided represent the Western Blot analysis of inhibitory effects of ET_A _receptor inhibitor BQ123 and MEK inhibitor U0126 on ET-1-induced phosphorylation of ERK1/2. 24 h Serum-starved cells were stimulated with 10 nM of ET-1 for 10 min after cells were treated with inhibitors for 30 min. Phosphorylated ERK1/2 activity was determined by western blot with an anti-phospho-ERK1/2 antibody, and presented as a relative extent to the level of β-actin. A, bar graph shows inhibitory effects of 5 μM of ET_A_/ET_B _receptor inhibitors on phosphorylated ERK1/2 activity induced by ET-1. B, bar graph shows inhibitory effect of 1 μM of U0126 on phosphorylated ERK1/2 activity induced by ET-1. The upper panels of A and B indicate representative autoradiographs of western blot showing phosphorylated ERK1/2 and β-actin. Data represent mean ± S.E.M. * p < 0.05, ** p < 0.01 compared with the ET-1-stimulated states after DMSO treatment. p = phosphorylation, ns = non-significant. Click here for file

Additional file 2 **Effect of ET-1 on activation of ERK1/2 in HASMCs in the absence of external Ca^2+^**. The data provided represent the immunofluorescence analysis of ET-1-induced phosphorylation of ERK1/2 in the absence of external Ca^2+^by replacing culture medium with PBS. Serum-starved cells were placed in the presence or absence of external Ca^2+ ^for 3 min by replacing culture medium with PBS plus 1 mM EGTA prior to addition of ET-1. Phosphorylated ERK1/2 was determined at 10 min after the addition of 10 nM of ET-1 by immunofluorescence with an anti-phospho-ERK1/2 antibody. The bar graph shows effect of ET-1 on phosphorylated ERK1/2 in the absence of extracellular Ca^2+^. The fluorescence intensities of phosphorylated ERK1/2 are expressed relative to the quiescent state in the presence of external Ca^2+^. The upper panel indicates representative images of immunofluorescence showing the phosphorylated ERK1/2 from samples given the different treatments. Data represent the mean ± S.E.M. *** p < 0.001. ns = non-significant. Click here for file

Additional file 3 **The Ca^2+ ^chelator EGTA abolished thapsigargin-induced activation of ERK1/2 in ET-1 untreated starved cells**. The data provided represent the immunofluorescence analysis of inhibitory effect of the Ca^2+ ^chelator EGTA on extracellular Ca^2+^influx through thapsigargin-induced store-operated Ca^2+ ^channels. Serum-starved cells were treated with 1 μM of thapsigargin with or without 5 μM of EGTA for 15 min. Phosphorylated ERK1/2 was determined by immunofluorescence with an anti-phospho-ERK1/2 antibody. The bar graph shows effect of thapsigargin on phosphorylated ERK1/2 in the presence or in the absence of EGTA. The upper panel indicates representative images of immunofluorescence showing the phosphorylated ERK1/2 from samples given the different treatments. Data represent mean ± S.E.M. *** p < 0.001 compared with the vehicle value. Click here for file

Additional file 4 **Effects of the inhibitors used in the present study on the activities of ERK1/2 in ET-1 untreated cells**. The data provided represent the immunofluorescence analysis of the stability of fluorescence intensity after cells were treated with inhibitors compared with vehicle treatment. Serum-starved cells were treated with variety of inhibitors indicated or DMSO for 30 min. Phosphorylated ERK1/2 was determined by immunofluorescence with an anti-phospho-ERK1/2 antibody. The bar graph shows no significant effects of the inhibitors on phosphorylated ERK1/2 in ET-1 untreated control cells. The upper panel indicates representative images of immunofluorescence showing the phosphorylated ERK1/2 from samples treated with different inhibitors. Data represent mean ± S.E.M. Click here for file

## References

[B1] Marasciulo FL, Montagnani M, Potenza MA (2006). Endothelin-1: the yin and yang on vascular function. Curr Med Chem.

[B2] Schneider MP, Boesen EI, Pollock DM (2007). Contrasting actions of endothelin ET(A) and ET(B) receptors in cardiovascular disease. Annu Rev Pharmacol Toxicol.

[B3] Masaki T (2004). Historical review: Endothelin. Trends Pharmacol Sci.

[B4] Adner M, Uddman E, Cardell LO, Edvinsson L (1998). Regional variation in appearance of vascular contractile endothelin-B receptors following organ culture. Cardiovasc Res.

[B5] Cramer H, Schmenger K, Heinrich K, Horstmeyer A, Böning H, Breit A, Piiper A, Lundstrom K, Müller-Esterl W, Schroeder C (2001). Coupling of endothelin receptors to the ERK/MAP kinase pathway. Roles of palmitoylation and G(alpha)q. Eur J Biochem.

[B6] Gohla A, Schultz G, Offermanns S (2000). Role for G(12)/G(13) in agonist-induced vascular smooth muscle cell contraction. Circ Res.

[B7] Pearson G, Robinson F, Beers Gibson T, Xu BE, Karandikar M, Berman K, Cobb MH (2001). Mitogen-activated protein (MAP) kinase pathways: regulation and physiological functions. Endocr Rev.

[B8] Liu B, Ryer EJ, Kundi R, Kamiya K, Itoh H, Faries PL, Sakakibara K, Kent KC (2007). Protein kinase C-delta regulates migration and proliferation of vascular smooth muscle cells through the extracellular signal-regulated kinase 1/2. J Vasc Surg.

[B9] Zhan Y, Kim S, Izumi Y, Izumiya Y, Nakao T, Miyazaki H, Iwao H (2003). Role of JNK, p38, and ERK in platelet-derived growth factor-induced vascular proliferation, migration, and gene expression. Arterioscler Thromb Vasc Biol.

[B10] D'Angelo G, Adam LP (2002). Inhibition of ERK attenuates force development by lowering myosin light chain phosphorylation. Am J Physiol Heart Circ Physiol.

[B11] Dessy C, Kim I, Sougnez CL, Laporte R, Morgan KG (1998). A role for MAP kinase in differentiated smooth muscle contraction evoked by alpha-adrenoceptor stimulation. Am J Physiol.

[B12] Luo G, Jamali R, Cao YX, Edvinsson L, Xu CB (2006). Vascular endothelin ET(B) receptor-mediated contraction requires phosphorylation of ERK1/2 proteins. Eur J Pharmacol.

[B13] Whitmarsh AJ, Davis RJ (1996). Transcription factor AP-1 regulation by mitogen-activated protein kinase signal transduction pathways. J Mol Med.

[B14] Yogi A, Callera GE, Montezano AC, Aranha AB, Tostes RC, Schiffrin EL, Touyz RM (2007). Endothelin-1, but not Ang II, activates MAP kinases through c-Src independent Ras-Raf dependent pathways in vascular smooth muscle cells. Arterioscler Thromb Vasc Biol.

[B15] Lucchesi PA, Bell JM, Willis LS, Byron KL, Corson MA, Berk BC (1996). Ca(2+)-dependent mitogen-activated protein kinase activation in spontaneously hypertensive rat vascular smooth muscle defines a hypertensive signal transduction phenotype. Circ Res.

[B16] Eguchi S, Matsumoto T, Motley ED, Utsunomiya H, Inagami T (1996). Identification of an essential signaling cascade for mitogen-activated protein kinase activation by angiotensin II in cultured rat vascular smooth muscle cells. Possible requirement of Gq-mediated p21ras activation coupled to a Ca^2+^/calmodulin-sensitive tyrosine kinase. J Biol Chem.

[B17] Eguchi S, Numaguchi K, Iwasaki H, Matsumoto T, Yamakawa T, Utsunomiya H, Motley ED, Kawakatsu H, Owada KM, Hirata Y, Marumo F, Inagami T (1998). Calcium-dependent epidermal growth factor receptor transactivation mediates the angiotensin II-induced mitogen-activated protein kinase activation in vascular smooth muscle cells. J Biol Chem.

[B18] McNair LL, Salamanca DA, Khalil RA (2004). Endothelin-1 promotes Ca^2+ ^antagonist-insensitive coronary smooth muscle contraction via activation of epsilon-protein kinase C. Hypertension.

[B19] Lee HM, Won KJ, Kim J, Park HJ, Kim HJ, Roh HY, Lee SH, Lee CK, Kim B (2007). Endothelin-1 induces contraction via a Syk-mediated p38 mitogen-activated protein kinase pathway in rat aortic smooth muscle. J Pharmacol Sci.

[B20] Berridge MJ, Bootman MD, Roderick HL (2003). Calcium signalling: dynamics, homeostasis and remodelling. Nat Rev Mol Cell Biol.

[B21] Chang WC, Di Capite J, Singaravelu K, Nelson C, Halse V, Parekh AB (2008). Local Ca^2+ ^influx through Ca^2+ ^release-activated (CRAC) channels stimulates production of an intracellular messenger and an intercellular pro-inflammatory signal. J Biol Chem.

[B22] Kim S, Iwao H (2000). Molecular and cellular mechanisms of angiotensin II-mediated cardiovascular and renal diseases. Pharmacol Rev.

[B23] Lee KH, Lim S, Kang SM, Kim DH, Cho HK, Chung JH, Kwon HM, Chung H, Lee H, Jang Y, Hwang KC (2004). Antiproliferative mechanisms of raxofelast (IRFI-016) in H2O2-stimulated rat aortic smooth muscle cells. Eur J Pharmacol.

[B24] Cui MZ, Zhao G, Winokur AL, Laag E, Bydash JR, Penn MS, Chisolm GM, Xu X (2003). Lysophosphatidic acid induction of tissue factor expression in aortic smooth muscle cells. Arterioscler Thromb Vasc Bio.

[B25] Zeidan A, Broman J, Hellstrand P, Swärd K (2003). Cholesterol dependence of vascular ERK1/2 activation and growth in response to stretch: role of endothelin-1. Arterioscler Thromb Vasc Biol.

[B26] Lüscher TF, Barton M (2000). Endothelins and endothelin receptor antagonists: therapeutic considerations for a novel class of cardiovascular drugs. Circulation.

[B27] Yoshizumi M, Kim S, Kagami S, Hamaguchi A, Tsuchiya K, Houchi H, Iwao H, Kido H, Tamaki T (1998). Effect of endothelin-1 (1–31) on extracellular signal-regulated kinase and proliferation of human coronary artery smooth muscle cells. Br J Pharmacol.

[B28] Hazzalin CA, Mahadevan LC (2002). MAPK-regulated transcription: a continuously variable gene switch?. Nat Rev Mol Cell Biol.

[B29] Davenport AP, O'Reilly G, Kuc RE (1995). Endothelin ET_A _and ET_B _mRNA and receptors are expressed by smooth muscle in the human vasculature: majority of the ET_A _subtype. Br J Pharmacol.

[B30] Maguire JJ, Davenport AP (1995). ET_A _receptor-mediated constrictor responses to endothelin peptides in human blood vessels in vitro. Br J Pharmacol.

[B31] Haynes WG, Strachan FE, Webb DJ (1995). Endothelin ET_A _and ET_B _receptors cause vasoconstriction of human resistance and capacitance vessels in vivo. Circulation.

[B32] Davenport AP (2002). Update on endothelin receptor nomenclature. Pharmacol Rev.

[B33] Gregan B, Jürgensen J, Papsdorf G, Furkert J, Schaefer M, Beyermann M, Rosenthal W, Oksche A (2004). Ligand-dependent differences in the internalization of endothelin A and endothelin B receptor heterodimers. J Biol Chem.

[B34] Sakamoto A, Yanagisawa M, Sawamura T, Enoki T, Ohtani T, Sakurai T, Nakao K, Toyo-oka T, Masaki T (1993). Distinct subdomains of human endothelin receptors determine their selectivity to endothelinA-selective antagonist and endothelinB-selective agonists. J Biol Chem.

[B35] Harada N, Himeno A, Shigematsu K, Sumikawa K, Niwa M (2002). Endothelin-1 binding to endothelin receptors in the rat anterior pituitary gland: possible formation of an ETA-ETB receptor heterodimer. Cell Mol Neurobiol.

[B36] Pérez-Rivera AA, Fink GD, Galligan JJ (2005). Vascular reactivity of mesenteric arteries and veins to endothelin-1 in a murine model of high blood pressure. Vascul Pharmacol.

[B37] Ginnan R, Singer HA (2002). CaM kinase II-dependent activation of tyrosine kinases and ERK1/2 in vascular smooth muscle. Am J Physiol Cell Physiol.

[B38] Dulin NO, Niu J, Browning DD, Ye RD, Voyno-Yasenetskaya T (2001). Cyclic AMP-independent activation of protein kinase A by vasoactive peptides. J Biol Chem.

[B39] Kolch W (2005). Coordinating ERK/MAPK signalling through scaffolds and inhibitors. Nat Rev Mol Cell Biol.

[B40] Marshall CJ (1996). Ras effectors. Curr Opin Cell Biol.

[B41] Vanhaesebroeck B, Leevers S, Ahmadi K, Timms J, Katso R, Driscoll P, Woscholski R, Parker P, Waterfield M (2001). Synthesis and function of 3-phosphorylated inositol lipids. Annu Rev Biochem.

[B42] Force T, Kuida K, Namchuk M, Parang K, Kyriakis JM (2004). Inhibitors of protein kinase signaling pathways: emerging therapies for cardiovascular disease. Circulation.

[B43] Pollock DM, Keith TL, Highsmith RF (1995). Endothelin receptors and calcium signaling. FASEB J.

[B44] Kawanabe Y, Hashimoto N, Masaki T (2002). Ca(2+) channels involved in endothelin-induced mitogenic response in carotid artery vascular smooth muscle cells. Am J Physiol Cell Physiol.

[B45] Abraham ST, Benscoter HA, Schworer CM, Singer HA (1997). A role for/calmodulin-dependent protein kinase II in the mitogen-activated protein kinase signaling cascade of cultured rat aortic vascular smooth muscle cells. Circ Res.

[B46] Ginnan R, Pfleiderer PJ, Pumiglia K, Singer HA (2004). PKC-delta and CaMKII-delta 2 mediate ATP-dependent activation of ERK1/2 in vascular smooth muscle. Am J Physiol Cell Physiol.

[B47] Kawanabe Y, Hashimoto N, Masaki T (2002). Extracellular Ca2+ influx and endothelin-1-induced intracellular mitogenic cascades in rabbit internal carotid artery vascular smooth muscle cells. J Cardiovasc Pharmacol.

[B48] Adner M, Cantera L, Ehlert F, Nilsson L, Edvinsson L (1996). Plasticity of contractile endothelin-B receptors in human arteries after organ culture. Br J Pharmacol.

