# Supplementary figures and images for: Role of ERK/MAPK in endothelin receptor signaling in human aortic smooth muscle cells
Source: BMC Cell Biol. 2009 Jul 3;10:52. doi: 10.1186/1471-2121-10-52 (PMC2715373; doi:10.1186/1471-2121-10-52)

A

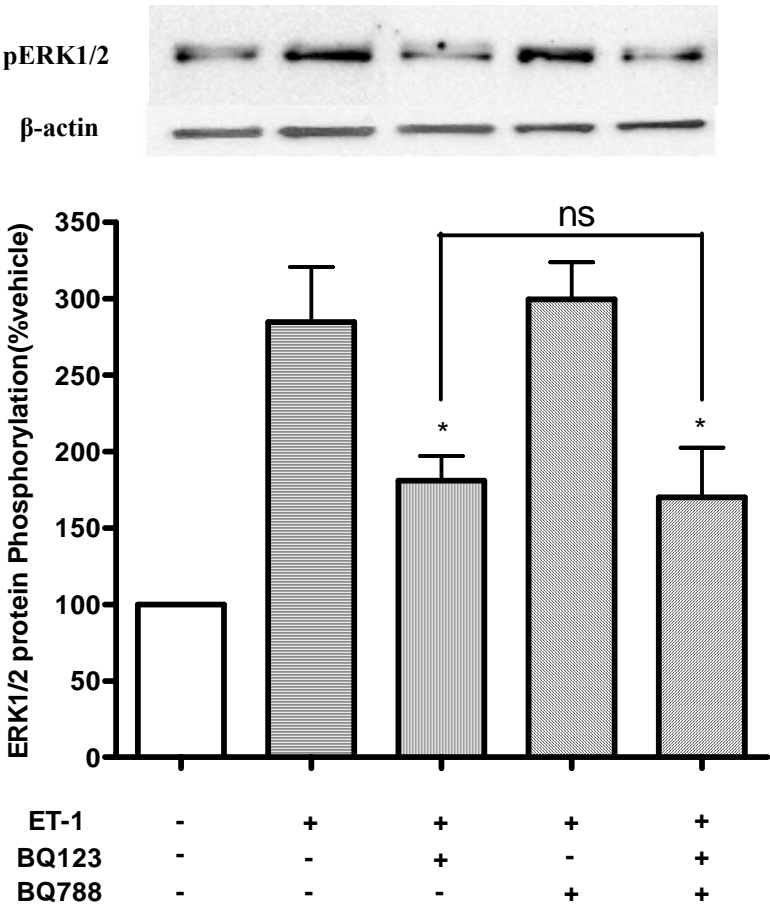

B

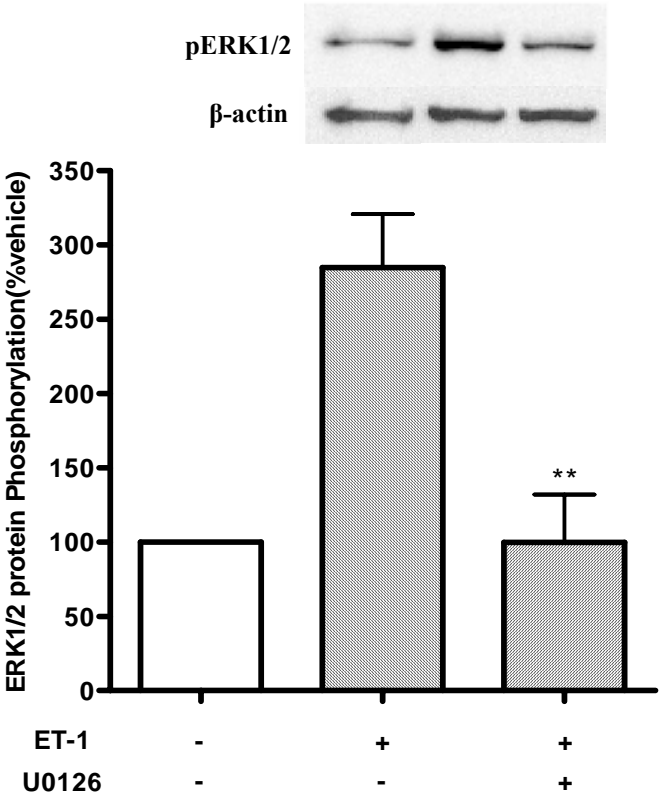

Supplement: Additional file 1 — Inhibitory effects of BQ123 and U0126 on pERK1/2 activity in HASMCs. The data provided represent the Western Blot analysis of inhibitory effects of ETA receptor inhibitor BQ123 and MEK inhibitor U0126 on ET-1-induced phosphorylation of ERK1/2. 24 h Serum-starved cells were stimulated with 10 nM of ET-1 for 10 min after cells were treated with inhibitors for 30 min. Phosphorylated ERK1/2 activity was determined by western blot with an anti-phospho-ERK1/2 antibody, and presented as a relative extent to the level of β-actin. A, bar graph shows inhibitory effects of 5 μM of ETA/ETB receptor inhibitors on phosphorylated ERK1/2 activity induced by ET-1. B, bar graph shows inhibitory effect of 1 μM of U0126 on phosphorylated ERK1/2 activity induced by ET-1. The upper panels of A and B indicate representative autoradiographs of western blot showing phosphorylated ERK1/2 and β-actin. Data represent mean ± S.E.M. * p < 0.05, ** p < 0.01 compared with the ET-1-stimulated states after DMSO treatment. p = phosphorylation, ns = non-significant. [file 1471-2121-10-52-S1.pdf]

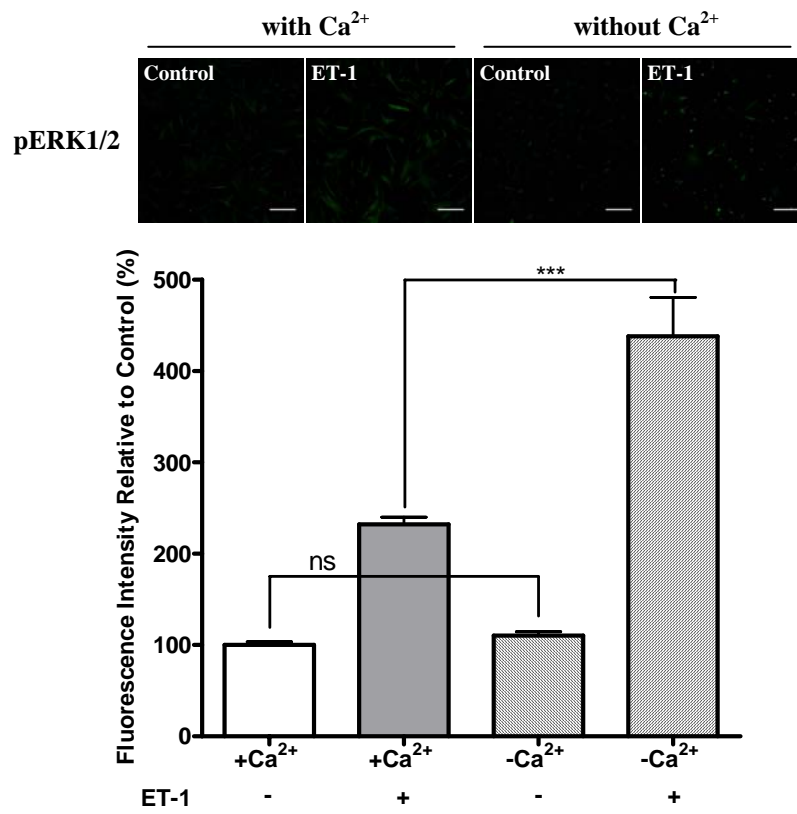

Supplement: Additional file 2 — Effect of ET-1 on activation of ERK1/2 in HASMCs in the absence of external Ca2+. The data provided represent the immunofluorescence analysis of ET-1-induced phosphorylation of ERK1/2 in the absence of external Ca2+by replacing culture medium with PBS. Serum-starved cells were placed in the presence or absence of external Ca2+ for 3 min by replacing culture medium with PBS plus 1 mM EGTA prior to addition of ET-1. Phosphorylated ERK1/2 was determined at 10 min after the addition of 10 nM of ET-1 by immunofluorescence with an anti-phospho-ERK1/2 antibody. The bar graph shows effect of ET-1 on phosphorylated ERK1/2 in the absence of extracellular Ca2+. The fluorescence intensities of phosphorylated ERK1/2 are expressed relative to the quiescent state in the presence of external Ca2+. The upper panel indicates representative images of immunofluorescence showing the phosphorylated ERK1/2 from samples given the different treatments. Data represent the mean ± S.E.M. *** p < 0.001. ns = non-significant. [file 1471-2121-10-52-S2.pdf]

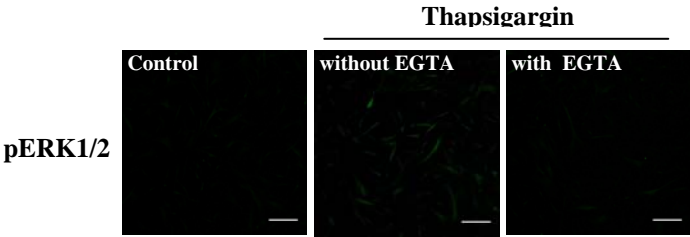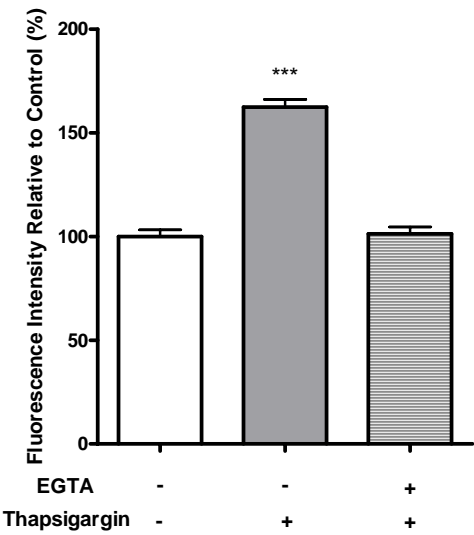

Supplement: Additional file 3 — The Ca2+ chelator EGTA abolished thapsigargin-induced activation of ERK1/2 in ET-1 untreated starved cells. The data provided represent the immunofluorescence analysis of inhibitory effect of the Ca2+ chelator EGTA on extracellular Ca2+influx through thapsigargin-induced store-operated Ca2+ channels. Serum-starved cells were treated with 1 μM of thapsigargin with or without 5 μM of EGTA for 15 min. Phosphorylated ERK1/2 was determined by immunofluorescence with an anti-phospho-ERK1/2 antibody. The bar graph shows effect of thapsigargin on phosphorylated ERK1/2 in the presence or in the absence of EGTA. The upper panel indicates representative images of immunofluorescence showing the phosphorylated ERK1/2 from samples given the different treatments. Data represent mean ± S.E.M. *** p < 0.001 compared with the vehicle value. [file 1471-2121-10-52-S3.pdf]

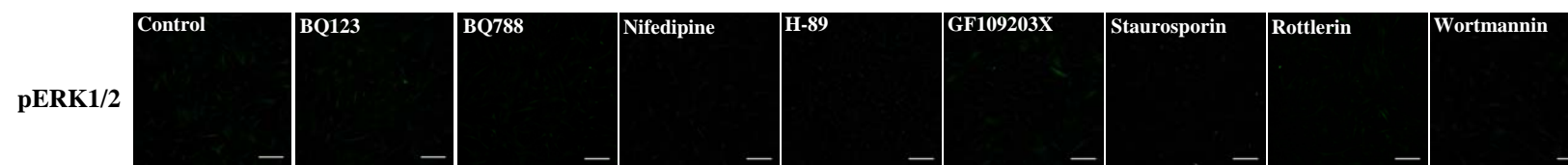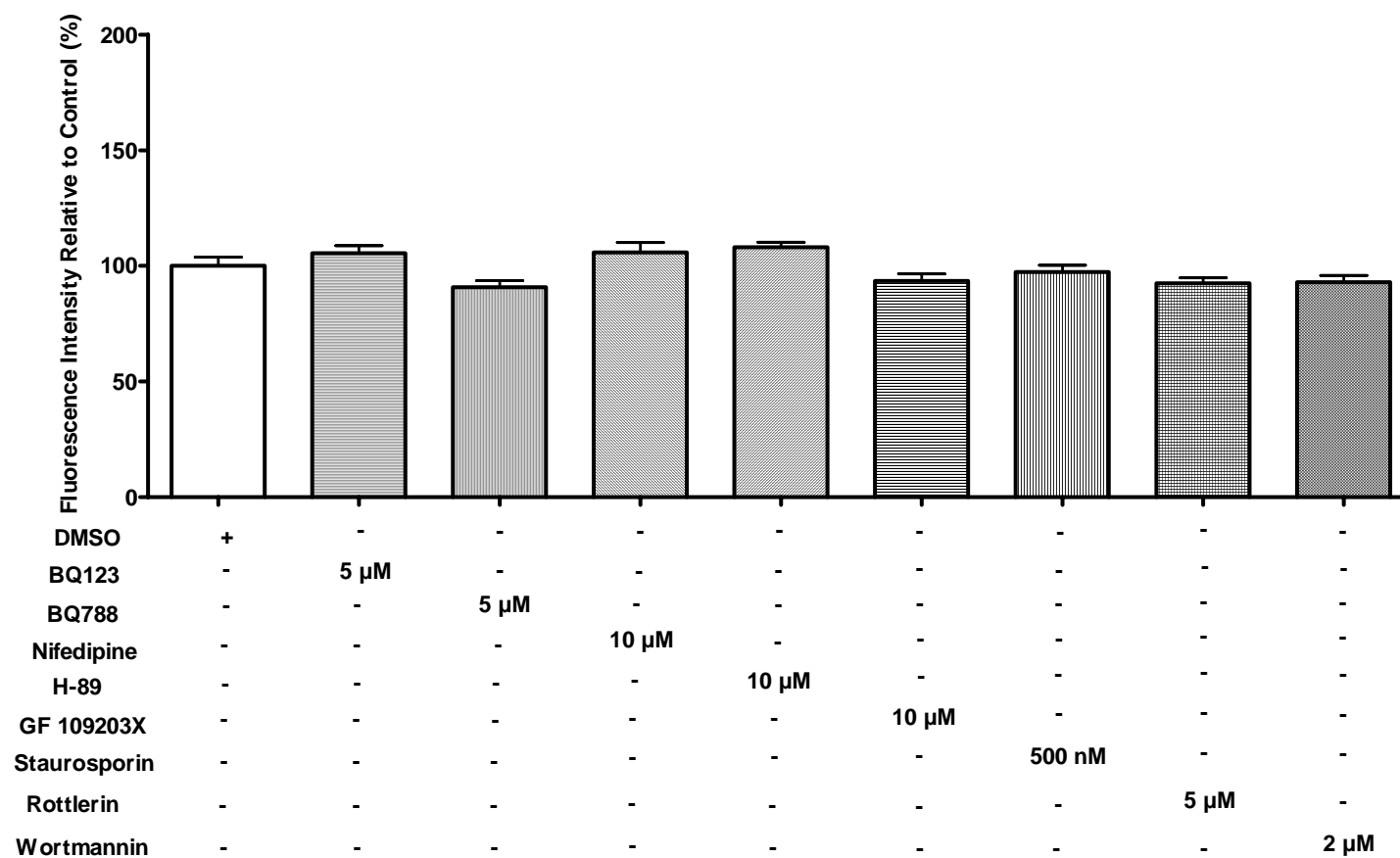

Supplement: Additional file 4 — Effects of the inhibitors used in the present study on the activities of ERK1/2 in ET-1 untreated cells. The data provided represent the immunofluorescence analysis of the stability of fluorescence intensity after cells were treated with inhibitors compared with vehicle treatment. Serum-starved cells were treated with variety of inhibitors indicated or DMSO for 30 min. Phosphorylated ERK1/2 was determined by immunofluorescence with an anti-phospho-ERK1/2 antibody. The bar graph shows no significant effects of the inhibitors on phosphorylated ERK1/2 in ET-1 untreated control cells. The upper panel indicates representative images of immunofluorescence showing the phosphorylated ERK1/2 from samples treated with different inhibitors. Data represent mean ± S.E.M. [file 1471-2121-10-52-S4.pdf]
